# Efficacy of a Novel Narrow Knife with Water Jet Function for Colorectal Endoscopic Submucosal Dissection

**DOI:** 10.1155/2017/5897369

**Published:** 2017-09-10

**Authors:** Naohisa Yoshida, Takashi Toyonaga, Takaaki Murakami, Ryohei Hirose, Kiyoshi Ogiso, Yutaka Inada, Rafiz Abdul Rani, Yuji Naito, Mitsuo Kishimoto, Yoshiko Ohara, Takeshi Azuma, Yoshito Itoh

**Affiliations:** ^1^Department of Molecular Gastroenterology and Hepatology, Graduate School of Medical Science, Kyoto Prefectural University of Medicine, Kyoto, Japan; ^2^Department of Endoscopy, Kobe University Hospital, Hyogo, Japan; ^3^Department of Surgical Pathology, Graduate School of Medical Science, Kyoto Prefectural University of Medicine, Kyoto, Japan; ^4^Division of Gastroenterology, Department of Internal Medicine, Graduate School of Medicine, Kobe University, Kobe, Japan

## Abstract

**Backgrounds:**

With respect to the knife's design in colorectal endoscopic submucosal dissection (ESD), diameter, water jet function, and electric power are important because these relate to efficient dissection. In this study, we analyzed a novel, narrow ball tip-typed ESD knife with water jet function (Flush knife BT-S, diameter: 2.2 mm, length: 2000 mm, Fujifilm Co., Tokyo, Japan) compared to a regular diameter knife (Flush knife BT, diameter: 2.6 mm, length: 1800 mm).

**Methods:**

In laboratory and clinical research, electric power, knife insertion time, vacuum/suction amount with knife in the endoscopic channel, and water jet function were analyzed. We used a knife 2.0 mm long for BT-S and BT knives.

**Results:**

The BT-S showed faster mean knife insertion time (sec) and better vacuum amount (ml/min) compared to the BT (insertion time: 16.7 versus 21.6, *p* < 0.001, vacuum amount: 38.0 versus 14.0, *p* < 0.01). Additionally, the water jet function of the BT-S was not inferior. In 39 colorectal ESD cases in two institutions, there were mean 4.7 times (range: 1–28) of knife insertion. Suction under knife happened 59% (23/39) and suction of fluid could be done in 100%.

**Conclusions:**

Our study showed that the narrow knife allows significantly faster knife insertion, better vacuum function, and effective clinical results.

## 1. Introduction

The number of colorectal endoscopic submucosal dissection (ESD) for various locations has increased exponentially all over the world [1–5]. In the initial beginning stages, the perforation rate was reported to be really high (10.4%), even for experts [6]. However, recent improvements of devices and strategies make colorectal ESD safer.

ESD knife is a particularly important key device for safe and accurate ESD. There are various ESD knives currently available such as the tip-shaped-type, the ball-tip-type, the blade-type, and the scissor-type knife [7]. With respect to the knife's design, diameter, length, water jet function, and needed electric power are important because it relates to the vacuum function, enables usage of long colonoscope, and ensures efficient dissection. Recently, a narrow and long ESD knife with water jet function has been developed. In this study, we analyzed this novel knife compared to a knife with a regular diameter and length.

## 2. Materials and Methods

**Figure 1:**
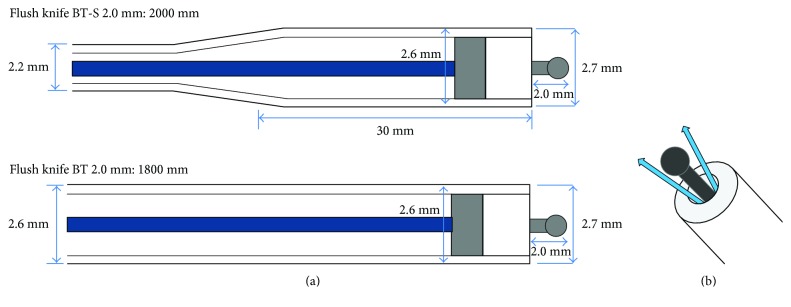
Shema of the Flush knife BT-S and the Flush knife BT. (a) The diameter of Flush knife BT-S is 2.2 mm, and it is slim designed but with only the front 3 cm tip part made slightly thick (2.6 mm) to anchor and ensure stable movement of knife during dissection. (b) Flush knife BT-S and BT have water jet function.

The novel narrow ESD knife (Flush knife BT-S 2.0 mm, diameter: 2.2 mm, Fujifilm Co., Tokyo, Japan) and the knife with regular diameter (Flush knife BT 2.0 mm, diameter: 2.6 mm, Fujifilm Co., Tokyo, Japan) were utilized in this study (Figure 1()) [8]. With respect to the diameter of the Flush knife BT-S, it is a slim design but with only the front 3 cm tip part made slightly thick (2.6 mm) to anchor and ensure stable movement of the knife during dissection. This thick part prevents that slim Flush knife BT-S moves a lot in an endoscopic channel during ESD, which is not good for accurate dissection. Additionally, the tip part of both knives was 2.7 mm. The length of Flush knife BT-S was 2000 mm and that of Flush knife BT was 1800 mm. Additionally, both knives had a ball-tip shape and water jet function.

With respect to laboratory research of the two knives, electric power, knife insertion time, vacuum function, and water jet function were analyzed. Electric power of each knife was supplied by an electrosurgical unit (VIO300D, Erbe Elektromedizin, Tubingen, Germany) and calculated by a specific analyzer (surgical analyzer: CDL1909A). The two electrical settings known as drycut mode (effect 4, 40 W) and forced coagulation mode (effect 2, 30 W) were used. Knife insertion time was calculated 30 times using a medium length colonoscope with 3.2 mm channel (EC-L600ZP, Fujifilm Co., Tokyo, Japan) for both knives. Vacuum function was also evaluated using the same colonoscope with each knife in the endoscopic channel. The vacuum amount of water in 1 minute was calculated 5 times at each three settings labeled as (1) without knife in the channel, (2) with the Flush knife BT-S, and (3) with the Flush knife BT. With respect to water jet function, we calculated the amount of water (ml/min) using each knife. In detail, we pressed the two knives to porcine submucosa and did injection in the condition of the knife closed. Then, we collected the flowed-out water which was injected. Then, we calculated the amount of water which flowed out. These two experiments were performed 5 times for each knife while utilizing a specific water jet system (JW-2, Fujifilm Co., Tokyo, Japan). Three settings of the water jet system such as low flow, middle flow, and high flow were used.

In an observational single-armed clinical research at two institutions, consecutive ESD cases using the Flush knife BT-S 2.0 mm performed in Kyoto Prefectural University of Medicine or Kobe University Hospital from March to June 2016 were enrolled. At both centers, the ESD were performed by five experienced endoscopists with a combined experience of more than 100 colorectal ESD cases between them. The indications for ESD included tumors that could not be resected by endoscopic mucosal resection (EMR), as well as those diagnosed as intramucosal cancer (Tis) or part of T1 cancer without risk of lymph node metastasis as evident by magnifying endoscopic examination with pit pattern observation, narrow band imaging (NBI), or blue laser imaging (BLI) [9, 10]. We analyzed the clinicopathological factors and outcomes including age, sex, tumor size, location, morphology, operation time, the rate of en bloc resection, histological diagnosis, and complications (perforation and postoperative hemorrhage) in all ESD cases. Additionally, the number of knife insertion during ESD and suction event with the knife in the endoscopic channel (suction under knife) was calculated. In the cases of suction under knife, whether water liquid could be vacuumed efficiently was evaluated.

The morphology of the tumor was divided into polypoid or nonpolypoid. The location of the tumor was identified according to the three segments: the right-sided colon (from the cecum to the transverse colon), the left-sided colon (from the descending to the sigmoid colon), and the rectum. In colorectal ESD, a lower gastrointestinal endoscope with a single channel (EC-L600ZP; diameter: 3.2 mm, Fujifilm Co., Tokyo, Japan) was used with a water jet system (JW-2, Fujifilm Co., Tokyo, Japan). We used Flush knife BT-S 2.0 mm.

## 3. Results

**Figure 2:**
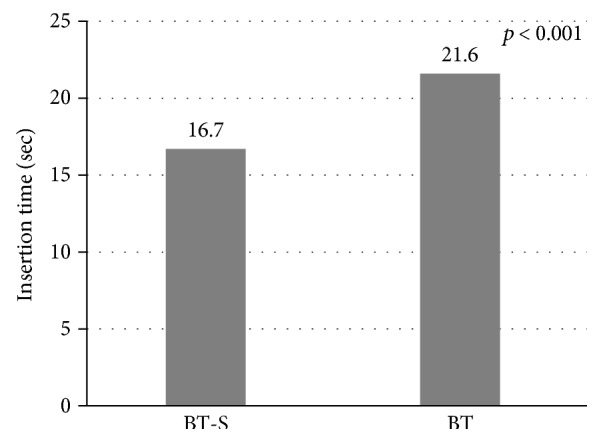
Knife insertion time of the Flush knife BT-S and the Flush knife BT for colonoscopy.

**Figure 3:**
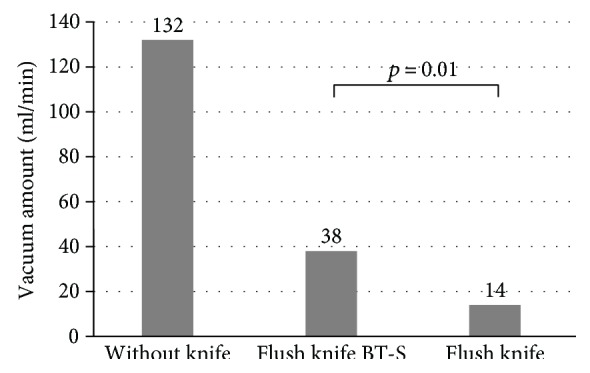
Vacuum amount of the Flush knife BT-S and the Flush knife BT in colonoscopy with 3.2 mm channel.

**Figure 4:**
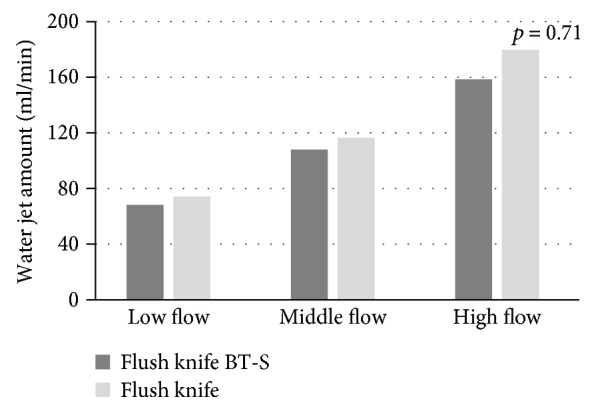
Water jet function to the submucosa of the porcine stomach in the Flush knife BT-S and the Flush knife BT.

In laboratory research, the values of electric power used in drycut mode of the Flush knife BT-S and BT were 282 mA, 40 W, 960 V, and 283 mA, 40 W, 965 V (not significant) (Table 1). On the other hand, those used in forced coagulation mode were 249 mA, 31 W, and 1305 V and 249 mA, 31 W, and 1310 V, respectively (not significant) (Table 1). The Flush knife BT-S showed faster knife insertion time (sec) compared to the Flush knife BT (knife insertion time: 16.7 versus 21.6, *p* < 0.001) (Figure 2()). Vacuum amount (ml/min) of the Flush knife BT-S in colonoscopy with 3.2 mm channel was significantly higher than that of the Flush knife BT (38.0 versus 14.0, *p* = 0.01) although those values were inferior than the amount without knife (132 ml/min) (Figure 3()). Water jet function was examined, and the mean amount of water (ml/min) to the submucosa of the porcine stomach in the Flush knife BT-S and the Flush knife BT was 108 and 116, respectively, when using the middle setting of water jet system (not significant) (Figure 4()).

In clinical research, 39 cases were analyzed in two institutions. Mean age of the group was 69.9 ± 10.7 years old and 27 were male (69.2%). Twenty-four (61.5%), 6 (15.4%), and 9 (23.1%) tumors were located in the right-sided colon, left-sided colon, and rectum, respectively (Table 2). While the mean tumor size was 32.0 ± 14.3 mm, 35 (89.7%) were nonpolypoid tumors. The rate of en bloc resection was 97.4% (38/39). Histologically, there were 2 (5.1%), 12 (30.8%), 17 (43.6%), and 8 (20.5%) cases of sessile-serrated adenoma and polyp (SSA/P), adenoma, Tis, and T1 cancers, respectively. Postoperative hemorrhage and perforation rate was at 2.6% (1/39) each. There were mean 4.7 times (range: 1–28) of ESD knife insertion among 39 ESD cases. Suction under knife happened in 23 (59%) out of 39 cases. In those cases, fluid and hemorrhage could be vacuumed in all 100% (23/23) of cases.

## 4. Discussion

Water jet function is useful not only for washing and submucosal injection but for the improvement of therapeutic results. A previous study about the comparison between the Flush knife and the Flex knife (no water jet function) showed mean operation time (min) was 61.0 and 87.3 (*p* = 0.02) [11]. Additionally, the Flush knife significantly reduced the number of device exchanges and the number of submucosal injections compared to the Flex knife. The Flush knife was subsequently modified for improvement as a ball-tip shape (Flush knife BT).

The effectiveness of submucosal injection is decided by two factors, which is the water jet amount and pressure. Theoretically, a thicker body/tube of knife is better in allowing for more liquid flow but at the expense of knife insertion time and vacuum or suction function under knife in the channel. A previous study showed a little decrease of water injection amount in the air with Flush knife BT-S (2–15 ml/min) compared to Flush knife [8]. However, in the current study using porcine stomach, the Flush knife BT-S did not show significant water jet function impairment compared to the Flush knife BT. We thought injection function in a laboratory research should be examined in the same situation of colorectal ESD. Thus, we pressed each knife to the porcine stomach and calculated the amount of water. Oppositely, a thin device is useful for efficient endoscopic vacuum. However, a thin device may not be stable in the channel and it prevents accurate operation of knife during ESD. The Flush knife BT-S is narrow, but with a thick part only at the front tip in order to stabilize the knife in the channel. Additionally, a thin knife can be easily bended, allowing a smooth and faster knife insertion. In the 39 clinical ESD cases, the mean number of knife insertion was 4.7 and the maximum number was 28. Thus, faster knife insertion using Flush knife BT-S is thought to be helpful for performing quick procedure and decreasing operator's stress. A thinner knife can be easily bended during insertion theoretically. This problem is resolved using the Flush knife BT-S. The main material component of the surrounding sheath of the Flush knife BT-S is polytetrafluoroethylene, and it is similar to the Flush knife BT, but the lumen of the Flush knife BT-S has been made narrower (2.0 mm to 1.45 mm) and making the knife harder. Recently, there have been other available ESD knives with a water jet function in Japan, although most of them are not yet to be available in the West.

The knife length is also important as a short knife disturbs operator's handling of endoscope and knife. Longer colonoscope is used regularly in the West, and as such, some of the Japanese endoscopic devices and accessories cannot be adopted because of short length. The Flush knife BT is good for upper endoscope and medium length colonoscope, but it is short for longer colonoscope. The Flush knife BT-S is 200 mm longer than the Flush knife BT.

With respect to vacuum function, our laboratory research showed significant difference between Flush knife BT-S and Flush knife BT. In the 39 clinical ESD cases using Flush knife BT-S, our data showed that 59% of the cases that needed suction under the knife in the channel for aspirating liquid and hemorrhage. We could vacuum fluid and hemorrhage could be vacuumed in all cases. Although the difference of Flush knife BT-S and BT is only 0.4 mm, however, this small but significant difference enabled us to suction fluid efficiently during ESD, improving time and ease of procedure.

A limitation of this study was its single-arm design and the small sample size. There are five size settings for Flush knife BT-S (1.0, 1.5, 2.0, 2.5, and 3.0 mm). We used only Flush knife 2.0 mm in laboratory research and clinical studies. We compared the Flush knife BT-S only with the Flush knife BT, not the other knives. According to the instruction manual for the waterjet system (JW-2), we used not saline but water for examining water jet function in the laboratory research, which we usually do not use in the clinical setting.

## 5. Conclusions

Unsuitable ESD knife requires higher mental concentration to maintain a safe and accurately performed procedure, inevitably increasing strain and slowing procedure speed. The Flush knife BT-S is one of the more recent novel designed purposely just for ESD procedures. Our study showed that the narrow and long ESD knife allows significantly faster knife insertion, better vacuum function, and effective clinical results.

## Figures and Tables

**Table 1:** The values of electric power in drycut mode and forced coagulation mode of the Flush knife BT-S and BT.

	Drycut effect4 40 W		Forced coagulation effect2 30 W	
	Flush knife BT-S	Flush knife BT	Flush knife BT-S	Flush knife BT
Electric current flow (mA)	282	283	249	249
Electric power (W)	40	40	31	31
Voltage (V)	960	965	1305	1310

**Table 2:** Clinicopathological results of ESD with the Flush knife BT-S in two institutions.

Case number	39
Age, mean ± SD	69.9 ± 10.3
Gender, male/female (%) (*n*)	69.2 (27)/30.8 (12)
Tumor size, mm, mean ± SD	32.0 ± 14.3
Tumor size ≥ 50 mm (%) (*n*)	15.4 (6)
Operator expert/nonexpert (%) (*n*)	71.8 (28)/28.2 (11)
Morphology (%) (*n*) Nonpolypoid/polypoid	89.7 (35)/10.3 (4)
Tumor location (%) (*n*) Right-sided/left-sided/rectum	61.5 (24)/15.4 (6)/23.1 (9)
Severe fibrosis (%) (*n*)	15.4 (6)
Difficult manipulation (%) (*n*)	35.9 (14)
Severe breathing movement (%) (*n*)	28.2 (11)
Two devices (%) (*n*)	43.6 (17)
Number of knife insertion, mean (range)	4.7 (1–28)
Suction case with knife (%) (*n*)	59.0 (23)
Mean operation time, min (range)	80.2 (19–210)
En bloc resection rate (%) (*n*)	97.4 (38)
Histological diagnosis (%) (*n*) AD/SSAP/Tis/T1	5.1 (2)/30.8 (12)/43.6 (17)/20.5 (8)
Perforation (%) (*n*)	2.6 (1)
Postoperative hemorrhage (%) (*n*)	2.6 (1)

Right-sided: cecum to transvers colon; left-sided: descending colon to sigmoid colon; Ad: adenoma; SSAP: sessile-serrated adenoma and polyp.
